# In How Many Ways Do We Hear?

**Published:** 1887-06

**Authors:** 


### IN HOW MANY WAYS DO WE HEAR?

Sound is a sensation caused by rapidly succeeding to-and-fro motions of the air, which touch points in the auricle, are reflected, touch the canal of the ear, and are then concentrated on the drum, or membrana tympani. Back and within this membrate is a delicate portion of bone, termed the handle of the malleus or hammer. Fitting into this upper end or head of the hammer is the incus, or anvil, united by a cog-like joint. The third bone is termed the stapes, or stirrup. This last bone, which is the most delicate and also the most important in its relations to every other bone, may be lost, and the drum-membrane gone, yet hearing will remain. It is attached to an oval membrane, which covers a depression in the bone, termed the oval window, being very movable, the membrane is larger than the foot of the stirrup.

The oval cavity is termed the vestibule, and from it proceed three small openings called the semi-circular canals. Forming a part of this complex yet beautiful apparatus, there is a cavity to which has been given the name of the cochlea, from its resemblance to a snail's shell. The cavity of the inner ear is filled with a liquid, in which are spread out the delicate fibers of the auditory nerve, or nerve of hearing. This nerve is the portio mollis of the seventh cranial or head nerves. As its Latin name indicates, it is very soft and impressible, and also liable to injury from many causes. It arises from the medulla oblongata, and the fourth ventricle of the brain enters the internal auditory canal, and is distributed to the labyrinth on a membrane within the bony cochlea. It is lined with epithelium of a velvet-like nature. A portion of this is termed the organ of Corti, and receives the termination of the auditory nerve. This organ of Corti consists of two rows of rod-like bodies, named the rods of Corti, and several rows of ciliated cells.

Sound is conducted to the membrana tympani, touching it with a delicate or powerful touch, being transferred to the chain of bones or ossicles. These movable semi-solid bodies vibrate, and carry the sound-waves to the fluid of the internal ear by means of the foot-plate of the stapes or stirrup, where they touch the fibers of the auditory nerve, being conveyed to the center of hearing in the brain.

These differences in vibrations make sounds higher or lower in pitch, loud or soft, simple or compound. Infinitely more complex are the vibrations produced by an orchestra with a chorus of human voices and solo singers associated. The mind fails in the effort to grasp the wave-form of the flood of complex vibrations that pours into the ear at every moment. The highest and lowest tones are heard; the qualities of the notes produced by the strings and wind instruments and the voice are all discernible. The theory offered by Helmholtz in explanation of this wonderful property of the sense of hearing is that the terminations of the nerves in our ears can analyze complex vibrations. It was maintained by Helmholtz and others that in the ear every audible tone throws into sympathetic vibration one or more of the nerve terminations, and that all complex tones are analyzed by them into simple pendular vibrations, which affect different nerve terminations according to their frequency of vibrations.

Although this theory is at the present day doubted by some careful observers, among the number Dr. W. Rutherford, Professor of Physiology of the University of Edinburgh, it has the acceptance of many able physicists.—*The Medical and Surgical Reporter*.

